# Prevalence and Characterization of Psychological Trauma in Patients with Fibromyalgia: A Cross-Sectional Study

**DOI:** 10.1155/2022/2114451

**Published:** 2022-11-30

**Authors:** Itxaso Gardoki-Souto, Diego Redolar-Ripoll, Marta Fontana, Bridget Hogg, María José Castro, Josep M. Blanch, Fabiola Ojeda, Aleix Solanes, Joaquim Radua, Alicia Valiente-Gómez, Roser Cirici, Víctor Pérez, Benedikt L. Amann, Ana Moreno-Alcázar

**Affiliations:** ^1^Forum Center Research Unit, Institute of Neuropsychiatry and Addictions (INAD), Parc de Salut Mar (PSMAR), Barcelona, Spain; ^2^Department of Psychiatry and Forensic Medicine, Autonomous Universtiy of Barcelona (UAB), Barcelona, Spain; ^3^Cognitive NeuroLab, Open University of Catalonia (UOC), Barcelona, Spain; ^4^Neuromodulation Unit, Brain 360 Institute, Barcelona, Spain; ^5^Hospital Del Mar Medical Research Institute (IMIM), Barcelona, Spain; ^6^Mental Health Networking Biomedical Research Centre (CIBERSAM), Institute of Health Carlos III, Madrid, Spain; ^7^Rheumatology Service, Parc de Salut Mar (PSMAR), Barcelona, Spain; ^8^August Pi I Sunyer Biomedical Research Institute (IDIBAPS), Barcelona, Spain; ^9^Karolinska Institute (KI), Stockholm, Sweden; ^10^King's College London, London, UK; ^11^Institute of Neuropsychiatry and Addictions (INAD), Parc de Salut Mar, Barcelona, Spain; ^12^Pompeu Fabra University (UPF), Barcelona, Spain; ^13^Department of Psychiatry and Psychotherapy, Ludwig Maximilian University Hospital, Munich, Germany; ^14^ISOMAE Institute of Neurosciences and Psychosomatic Psychology, Sant Cugat Del Vallés, Barcelona, Spain

## Abstract

**Background:**

Preliminary evidence suggests that psychological trauma, especially childhood trauma, is a risk factor for the onset of fibromyalgia (FM).

**Objective:**

The main objective of this study consisted of evaluating the prevalence and detailed characteristics of psychological trauma in a sample of patients with FM, the chronology of trauma across the lifespan, and its clinical symptoms. We also calculated whether childhood trauma could predict the relationship with different clinical variables.

**Method:**

Eighty-eight females underwent an interview to assess sociodemographic data, psychiatric comorbidities, level of pain, FM impact, clinical symptoms of anxiety, depression, insomnia, quality of life, and psychological trauma.

**Results:**

The majority of participants (71.5%) met the diagnostic criteria for current post-traumatic stress disorder (PTSD). Participants reported having suffered traumatic events throughout their lifespan, especially in childhood and early adolescence, in the form of emotional abuse, emotional neglect, sexual abuse, and physical abuse. Traumatic events predict both poor quality of life and a level of pain in adulthood. All patients showed clinically relevant levels of anxiety, depression, insomnia, suicidal thoughts, and pain, as well as somatic comorbidities and poor quality of life. Pain levels predicted anxiety, depression, dissociation, and insomnia symptoms. 84% of the sample suffered one or more traumatic events prior to the onset of pain.

**Conclusions:**

Our data highlight the clinical complexity of patients with FM and the role of childhood trauma in the onset and maintenance of FM, as well as the high comorbidity between anxiety, depression, somatic symptoms, and FM. Our data also supports FM patients experiencing further retraumatization as they age, with an extremely high prevalence of current PTSD in our sample. These findings underscore the need for multidisciplinary programs for FM patients to address their physical pain and their psychiatric and somatic conditions, pay special attention to the assessment of psychological trauma, and provide trauma-focused interventions. *Trial registration*: ClinicalTrials.gov NCT04476316. Registered on July 20th, 2020.

## 1. Introduction

Fibromyalgia (FM) is considered the second most common rheumatic disorder [1] and affects between 2% and 4% of the population worldwide, in the majority of cases females [2]. In addition, FM is often diagnosed alongside chronic fatigue syndrome (CFS), due to the overlap between their symptoms and their underlying biological mechanisms [3]. Although the etiology of FM remains unknown [4], several explanations have been proposed for the onset of FM, including genetic factors [5, 6], hormonal and immunological alterations [7], nutritional aspects [4], and abnormalities in central pain processing [4, 8] linked to the sensitization of the central nervous system (CNS) [4, 5, 9]. In fact, the sensitization of the CNS has been suggested as one of the main pathophysiological changes involved in FM, involving neurotransmitter imbalances, altered functional connectivity, and changes in the hypothalamic-pituitary-adrenal (HPA) axis, which influence the autonomic nervous system [5, 10, 11]. Interestingly, exposure to different chronic or acute stressors, including psychological trauma, has a significant impact on the dysregulation of the HPA axis [12]. Psychological trauma is defined as a stressful life event that causes distress and exceeds an individual's ability to integrate the emotions and cognitions involved in the experience [13]. These events happen in an unexpected and uncontrollable manner, putting the individual's physical and psychological integrity at risk, exceeding their coping resources and leading to responses of helplessness and fear [14]. In some cases, exposure to one or more traumatic events can result in a diagnosis of PTSD [15, 16] or the presence of traumatic symptoms that cause discomfort without meeting the necessary criteria for a PTSD diagnosis [17]. Trauma exposure can happen throughout the entire lifespan of a person, but if it occurs during childhood, its psychopathological and somatic consequences are severe due to the vulnerability of the CNS during infancy and adolescence [18,19]. In this sense, childhood trauma or adverse childhood experiences (ACEs) encompasses various forms of physical and emotional abuse or neglect, sexual abuse, bullying, or household dysfunction experienced in childhood. Along these lines, several studies have investigated the relationship between childhood trauma and FM [5, 20–22], and have found a strong association. More specifically, these studies have shown that women diagnosed with FM score higher on the childhood trauma questionnaire (CTQ) as compared to controls [23, 24] or other medical conditions such as migraine, rheumatoid arthritis, and myofascial pain syndrome [23, 25–27]. Further studies showed a higher prevalence of emotional abuse [24, 25] and physical neglect [23] in patients with FM compared with controls, and higher scores in emotional abuse [25, 27], emotional neglect [27], physical neglect [27], and physical abuse [26], in comparison with patients affected by rheumatoid arthritis and migraine. All this data seems to indicate that childhood trauma can be considered a risk factor for the development of unexplained physical pain [28, 29], especially for FM.

Despite all the studies that highlight the relationship between childhood trauma and FM, few studies have collected in detail the chronology of the traumatic events to which these patients are exposed throughout their lifespan, from infancy to adulthood, as well as information on whether these events occurred mainly before or after the onset of pain. Likewise, many studies have focused on studying the correlation between trauma and pain. However, there is a lack of studies on possible predictor variables that explain the relationship between childhood trauma, pain, and the presence of psychiatric comorbidities, which are highly prevalent in this population and significantly affect the functionality and quality of life of the patients.

Our study aimed to analyze the prevalence and clinical characterization of traumatic events present in women with a principal diagnosis of FM and to what extent these events can predict pain levels, the presence and intensity of affective symptoms, psychiatric comorbidity, insomnia, and quality of life.

## 2. Materials and Methods

### 2.1. Participants of the Study

The study sample consisted of 90 female participants diagnosed with FM, who were consecutively derived from the rheumatology service (*n* = 42) and from the adult mental health services (*n* = 30) of the Parc de Salut Mar in Barcelona, Spain. Further participants (*n* = 18) from the catchment area learnt about the study through word of mouth or from local FM associations and contacted researchers to voluntarily enroll. The study was conducted between January 2020 and January 2021. All women were evaluated through an individual clinical interview that lasted approximately 1.5 hours and was conducted by two psychologists from the forum center research unit. In some cases, the assessments were carried out in person, but when participants were not able to attend the interviews because of COVID-19-related restrictions, clinical evaluations were carried out online.

Participants had to fulfill the following inclusion criteria: (1) women with an FM diagnosis based on a clinical interview aligned with the American college of rheumatology criteria [30], and (2) aged between 18 and 70 years. The exclusion criteria are as follows: (1) comorbid chronic inflammatory or autoimmune disorders; (2) neurodegenerative disorders, which can interfere in the development of the clinical interviews; (3) severe mental disorders, namely bipolar disorder, schizoaffective disorder, or schizophrenia. Of note, major depressive disorder and anxiety disorders were not excluded as exclusion criteria due to the high prevalence of these disorders in patients with FM; (4) present active suicidal ideation; and (5) dependence or substance abuse (except nicotine) in the previous month before the study enrolment. Taking into account these criteria of the 90 participants, two women were excluded because of the diagnosis of an inflammatory or autoimmune disorder; therefore, the final sample consisted of 88 women.

The investigation was developed in accordance with the latest version of the Helsinki Declaration and was approved by the ethics committee “Comité Ético de Investigación Clínica del Parc de Salut Mar” (reference number: 2020/9158). All participants gave written informed consent following a full explanation of the study before enrolling.

### 2.2. Clinical Outcomes and Assessment

In the clinical interview, the evaluators gathered the data following a previously designed case report form (CRF) for the study. The CRF collected sociodemographic variables such as age, race, educational level, marital status, employment status, psychiatric and somatic comorbidities, and pharmacological treatment. A series of validated scales were also administered for the evaluation of pain and FM impact, psychological trauma and associated symptoms, comorbid psychiatric disorders, clinical symptoms of depression and anxiety, insomnia, and quality of life. The scales are detailed as follows:

#### 2.2.1. Pain and FM Impact

1. Visual analog scale (VAS) for pain [31]: This scale consists of a straight horizontal line, ranging from 0 (no pain) to 10 (maximum pain), which assesses the pain severity at the moment of the clinical interview. Scores are interpreted as “no pain” (0–2), “mild pain” (2–4), “moderate pain” (4–6), “severe pain” (6–8), and “maximum pain” (8–10).
2. Pain disability index (PDI) [32]: This scale comprises 7 items referring to different aspects of life that can be affected by chronic pain: home and family responsibilities, recreation, social activities, occupation, sexual activity, and life support activities. Each item can be rated from 0 (no disability) to 10 (total disability), and the total score is obtained by summing all the answers. Since there is currently no validated Spanish version, this scale was translated from the English version by a bilingual researcher.
3. Fibromyalgia impact questionnaire [33], Spanish validation (S-FIQ) [34]: This 10-item scale assesses the impact of FM over the previous 7 days. The first item contains 11 questions, which are related to physical functioning; items 2 and 3 ask the person to answer the number of days they have been unable to work because of FM; and items from 4 to 10 are scaled items (0–10), in which the person rates work difficulty, pain, fatigue, morning tiredness, rigidity, anxiety, and depression. Higher scores signify lower levels of functioning.

#### 2.2.2. Psychological Trauma and Associated Symptoms

1. Chronology of traumatic events: This tool consists of a table, which gathers all the traumatic events reported by the participants over their lifespan according to their age when each occurred. The table is divided into five-year segments spanning from 0–5 up to 65–70 years. For each segment, participants are asked, “Do you remember having any traumatic or stressful experiences when you were in this age group?”. Answers are then coded using thematic analysis. Traumatic events were then subsequently categorized as one of the following: emotional abuse; physical abuse; sexual abuse; emotional neglect; physical neglect; parental violence; drug abuse by a family member; parental mental disorder; parental mental disorder; accident; physical violence in adulthood; rape, abuse or sexual attack; harassment or psychological maltreatment in adulthood; death of a loved one; abortion; surgery; imprisonment, detention, or kidnapping; illness of a first-degree relative; personal illness; pregnancy itself or pregnancy complications; personal separation or divorce; mobbing; economic problems; familiar conflicts; others (such as bullying, natural disasters, eviction, or migration processes), or no traumatic event.
2. Global assessment of post-traumatic stress questionnaire (EGEP-5) [35]: This 55-item scale assess PTSD according to DSM-V criteria over the month prior to the evaluation, based on the event from the chronology currently causing the greatest level of distress. It evaluates symptoms of intrusion, avoidance, alterations in cognition, mood, arousal, and reactivity, as well as duration and impact on functioning, and determines the presence or absence of PTSD, as well as specifying if depersonalization, derealization, and/or delayed expression are present.
3. Subjective unit of distress (SUD) [36]: this scale, ranging from 0 (no distress) to 10 (maximum distress), evaluates the level of subjective perturbation a person experiences when they bring to mind the traumatic event chosen in the EGEP-5 scale.
4. Holmes and Rahe social readjustment scale [37], Spanish validation [38]: This scale lists 43 possible stressful events, and participants must mark the events that have happened to them in the previous 12 months. Each event has a different score, and the global score is reached by summing all the scores of the events selected. Scores under 150 indicate low levels of stress, scores between 150 and 299 are suggestive of a 50% risk of stress-related disorders, and scores above 300 mean an 80% risk of suffering stress-related disorders.
5. Childhood trauma questionnaire (CTQ) [39], Spanish validation [40]: This self-applied scale evaluates the presence of childhood trauma with 30 items (CTQ-T), which can be answered on a 5-point Likert scale. The CTQ measures 5 types of childhood maltreatment: emotional (CTQ-EA), physical (CTQ-PA), sexual abuse (CTQ-SA), emotional (CTQ-EN), and physical neglect (CTQ-PN). The numerical answers correspond with responses ranging from “never true” to “very often true”, and the final score of each factor indicates severity in terms of “none to minimal”, “low to moderate”, “moderate to severe” and “severe to extreme”.
6. Dissociative experiences scale (DES) [41], Spanish validation [42]: This 28-item scale assesses the frequency of the presence of different dissociative experiences, excluding acute intoxication by substances. Items are rated from 0% to 100%. The total score is obtained by summing all item answers and dividing this result by 28. There are three different subscales, such as amnesia (DES-A), depersonalization/derealization (DES-DP), and dissociation (DES-D). A total score (DES-T) equal to or higher than 30 is interpreted as a dissociative disorder.
7. Somatoform dissociation scale-20 (SDQ-20) [43], Spanish validation [44]: This questionnaire evaluates the presence and intensity of 20 somatoform symptoms or experiences that happened during the last year. Participants are asked to rate the intensity of each experience on a 5-point Likert scale and whether there is a medical explanation for it. The total score is reached by summing the scores for each item, while the symptoms with no known cause are summed to reach the total of symptoms with no known cause.

#### 2.2.3. Diagnosis

1. FM diagnosis: FM diagnosis based on a clinical interview aligned with the American college of rheumatology criteria [30], which was carried out in all participants.
2. Psychiatric comorbidities: MINI neuropsychiatric international interview [45], Spanish validation [46]: The MINI structured interview evaluates the presence of psychiatric comorbidity according to DSM-IV criteria. In the present research project, only the following parts of the scale have been used for the assessment: major depressive episode, dysthymic episode, manic/hypomanic episode, panic disorder, agoraphobia, social phobia, obsessive-compulsive disorder, and generalized anxiety disorder. The remaining MINI components were not used because the data were covered by the inclusion and exclusion criteria (substance dependence, substance abuse, psychotic disorders, anorexia nervosa, and bulimia nervosa), were collected during the clinical interview (suicidality), or were assessed more exhaustively by other specific psychometric scales (PTSD).

#### 2.2.4. Clinical Symptoms, Insomnia, and Quality of Life Evaluation

1. Hospital anxiety and depression scale (HADS) [47], Spanish validation [48]: This self-administered scale measures the presence and intensity of anxious and depressive symptoms in the previous 7 days. It contains 14 items, seven for each of the subscales (HADS-A for anxiety and HADS-D for depression), which can be rated from 0 to 3. The sum total of the scores gives the overall score. Scores equal to or over 8 for each subscale can represent the presence of an anxiety or depressive disorder.
2. Athens insomnia scale (AIS) [49], Spanish validation [50]: This scale evaluates the presence of insomnia according to ICD-10 criteria. It evaluates disturbances in sleep over the previous week and consists of 8 items. The first five assess sleep induction, awakenings at night, final awakening, total sleep duration, and sleep quality, and the last three items refer to well-being, functioning capacity, and sleepiness during the day. All items can be rated from 0 to 3, providing a total score when summed between 0 and 24.
3. Satisfaction with life scale (SWLS) [51], Spanish validation [52]: This self-applied scale consists of 5 items evaluating the self-perception of satisfaction with one's life. Each item can be rated from 1 (totally disagree) to 5 (totally agree). Higher scores represent higher levels of satisfaction.

## 3. Data Analysis

### 3.1. Sample Size Calculation

Given that the main objective of this study was to investigate the relationship between childhood trauma (CTQ) and FM pain (VAS pain), we calculated the sample size needed to conduct a regression analysis between CTQ and VAS pain. According to the standard formula, the sample size required to detect small correlations (*R* = 0.30) with 80% statistical power is 84. However, researchers decided to include all candidates who were recruited between January 2020 and January 2021 who signed the informed consent and met inclusion criteria, even if the calculated number was exceeded.

### 3.2. Statistical Analysis

All analyses were performed using STATA statistics/data analysis, version 16.1 (StataCorp LLC, Texas, USA). Fitness to parametric assumptions was checked for all variables. To assess the normality of data distribution, the Shapiro–Wilk test was used.

First, regarding the descriptive analysis of the sociodemographic, medical, and clinical data (Tables 1 and 2), the arithmetic mean was used for quantitative variables and the proportion for categorical variables. In both cases, the standard error and confidence interval (95%) were calculated.

Second, simple linear regression has been used to analyze whether different types of child maltreatment (CTQ measures) could predict different clinical variables, namely anxiety and depression.

Third, we also used the simple linear regression model to analyze whether the scores obtained on the fibromyalgia pain and impact scales (VAS, PDI, and S-FIQ) could predict the clinical variables discussed above (HADS and AIS) and the patients' quality of life (SWLQ).

## 4. Results

### 4.1. Sample Characteristics

The mean age of the 88 participants in the sample was 51.41 years, with the majority being married or in a couple, and Caucasian. According to their employment status, a high number of them were on sick leave (*n* = 31, 35.2%), unemployed (*n* = 31, 15%), or unable to work due to mental health problems or other problems (*n* = 23, 26%) (see Table 1).

Regarding the medical data of the sample, other comorbid axis III diagnoses were common, with the most prevalent being chronic fatigue syndrome (60.2%), followed by osteoarthritis (30.6%). Participants were prescribed various pharmacological drugs, with the most frequent being anxiolytics/hypnotics (60.9%); then, serotonin-norepinephrine reuptake inhibitors (SNRIs) (49.4%), paracetamol (36.7%), opioids (mainly tramadol and tapentadol, 22.7% and 9.1%, respectively) and anti-inflammatory drugs (35.2%) (see Table 2).

According to the MINI, anxiety disorders (87.5% for generalized anxiety disorder, 63.6% for panic disorder, and 29.5% for social phobia/agoraphobia) and mood disorders (76.1% for major depressive disorder and 11.3% for dysthymia) were the most frequent psychiatric comorbidities. These results were confirmed by higher current scores in the HADS (anxiety MD = 14.34; SD = 0.39 versus depression MD = 12.04; SD = 0.46). Participants showed marked scores in the AIS (MD = 14.14; SD = 0.52) suggestive of sleep disturbances and an important degree of dissatisfaction with their lives according to the SWLS scores (MD = 11.78; SD = 0.49). In terms of suicidal behavior, 82.9% of the participants reported having had thoughts of suicide at some point in their lives, and 32.9% had carried out at least one suicide attempt in their lifetime, of which 4.5% used violent methods (e.g. physical self-harming) and 27.2% nonviolent methods (e.g. drug over-ingest), 13.6% suffered severe consequences (e.g. hospitalization or physical lesions), and 18.18% nonsevere consequences (resolved without the need to go to health services) (see Table 2).

As expected, subjective pain perception, measured with the VAS, revealed high levels of pain (MD = 6.57; SD = 0.23). Furthermore, we also found a marked degree of disability (PDI MD = 46.12; SD = 1.21) with a generally large negative impact of FM on daily functioning and quality of life (S-FIQ MD = 55.58; SD = 1.06). The average number of years between the onset of pain and the diagnosis of FM in the sample was 9.10 (onset of pain: *M* = 35.44, SD = 11.93; FM diagnosis: *M* = 44.55, SD = 8.50) (see Table 2).

The EGEP-5 scores revealed that 71.5% of the participants met the criteria for PTSD in the last month. In relation to the event chosen by each participant for the administration of this scale, 34 women selected an event that occurred in their childhood, 47 an event that occurred in adulthood, and this data was missing for 7 participants. The average score of subjective distress, measured via SUD, was 8.23 (from 0 to 10, SD = 0.26), indicating great psychological distress in relation to that event (see Table 3). Total DES scores revealed low-to-moderate levels of dissociative symptoms in our sample (MD = 24.72; SD = 1.34); however, the dissociation subscale of dissociative experiences obtained scores above the cut-off point (MD = 36.26; SD = 1.86). Somatoform dissociative symptoms, measured by the SDQ-20, confirmed the presence of a strong somatization tendency in the sample (SD = 39.45; SD = 1.11), having an average of 4.76 symptoms with no known cause (SD = 0.3) (see Table 3).

Our FM sample experienced low-to-moderate levels of all types of child maltreatment in the CTQ, with both emotional abuse (63.6%) and emotional neglect (62.5%) being the most frequent maltreatment, followed by sexual abuse (48.8%), physical neglect (40.9%), and physical abuse (31.8%). Of note, minimization and denial in the CTQ were controlled for. Furthermore, patients showed an average of 7.64 (SD = 0.42) stressful events that happened during the last year and a high total score on the Holmes and Rahe scale (MD = 224.52; SD = 13.16), which is suggestive of a 50% risk of developing stress-related disorders (see Table 3).

**Figure 1:**
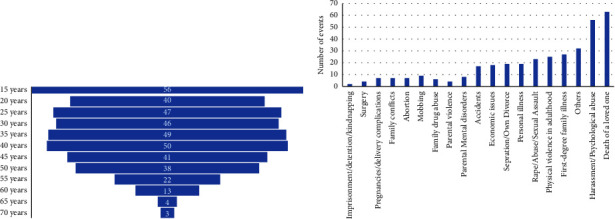
(a) Number of traumatic events per age (*n* = 77), and (b) typology of traumatic events throughout lifespans (*n* = 77).

**Figure 2:**
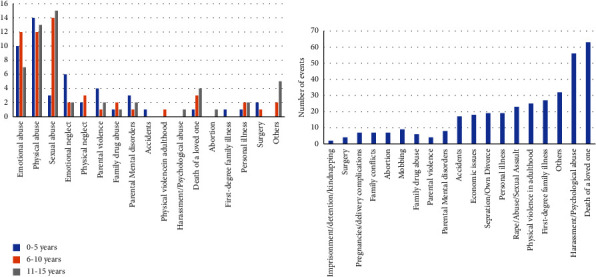
Number and typology of traumatic events (a) between 0 and 15 years (*n* = 77), and (b) between 16 and 70 years (*n* = 77).

Regarding the chronology of traumatic events, the highest number of these events occurred in the stages of second childhood and early adolescence. However, a high number of traumatic events were also reported during the first year of life (see Figure 1(a)). From Figure, it can also be gathered that traumatic events in this population begin to occur at very early ages and continue throughout all lifespans, well into late adulthood or older age, changing the typology of the exposed traumatic event (see Figure 1(b)). The most prevalent traumatic events to occur between 0 and 15 years were physical abuse (*n* = 39), sexual abuse (*n* = 32), and emotional abuse (*n* = 29) (see Figure 2(a)), while the most prevalent type of traumatic event in the sample after childhood and adolescence was the death of a loved one (*n* = 71), followed by harassment or psychological abuse in adulthood (*n* = 57), and other experiences grouped in the category “other traumatic events” (*n* = 39) (see Figure 2(b)). Interestingly, 84% of the sample suffered from one or more types of traumatic events prior to the onset of pain.

### 4.2. Simple Linear Regression

All linear regression results can be gathered from Table 4. Hereby, 3 types of child maltreatment (emotional abuse (CTQ-EA : *F* (1, 85) = 6.30; *p* = 0.0140), emotional neglect (CTQ-EN : *F* (1, 86) = 12.94; *p* = 0.0005), and physical neglect (CTQ-PN : *F* (1, 85) = 6.10; *p* = 0.0155) predicted low quality of life (high scores on the SWLS) in our sample. The same was true for the total score obtained on the CTQ (CTQ-T; *F* (1, 85) = 8.27; *p* = 0.0051) and two measures of pain, the PDI and S-FIQ (*F* (1, 85) = 8.69; *p* = 0.0041 and *F* (1, 85) = 8.08; *p* = 0.0056), respectively.

In addition, linear regression analysis also showed that higher scores in physical neglect significantly predicted higher levels of pain in the VAS (CTQ-PN: *F* (1, 86) = 4.12; *p* = 0.0455).

On the other hand, linear regression showed that higher scores in the pain measures and FM impact on quality of life (VAS, PDI, and S-FIQ) significantly predicted the presence and intensity of depressive and anxious symptomatology. More specifically, the VAS significantly predicted both depressive (HADS-D: *F* (1, 86) = 3.99; *p* = 0.0489) and anxious (HADS-A: *F* (1, 86) = 10.08; *p* = 0.0021) symptomatology, which was also true for the PDI (HADS-D: *F* (1, 86) = 31.14; *p* = 0.0000); HADS-A: *F* (1, 86) = 22.58; *p* = 0.0000) and S-FIQ scales (HADS-D: *F* (1, 85) = 12.39; *p* = 0.0007); HADS-A: *F* (1, 85) = 27.16; *p* = 0.0000).

Likewise, the scores in the pain and in the FM impact (VAS, PDI, and S-FIQ) also significantly predicted the degree of dissociative experiences (DES). VAS significantly predicted both the total score (DES-T: *F* (1, 86) = 4.31; *p* = 0,0410) and the scores obtained in dissociation (DES-D: *F* (1, 84) = 4.30; *p* = 0.0412). The PDI significantly predicted the total score (DES-T: *F* (1, 84) = 10.50; *p* = 0.0017) and the scores obtained in amnesia (DES-A: *F* (1, 85) = 6.27; *p* = 0.0142), depersonalization (DES-DP: *F* (1, 85) = 6.29; *p* = 0.0140), and dissociation (DES-D: *F* (1, 85) = 7.56; *p* = 0.0073) subscales. The same was true for the S-FIQ (DES-T: *F* (1, 83) = 11.83; *p* = 0.0000), DES-A: *F* (1, 83) = 8.61; *p* = 0.0043), DES-DP: *F* (1, 83) = 5.08; *p* = 0.0268), DES-D: *F* (1, 83) = 10.25; *p* = 0.0019).

Furthermore, linear regression analysis was also suggestive that patient scores on pain and FM impact (VAS, PDI, and S-FIQ) also significantly predicted sleep disturbance (AIS: *F* (1, 86) = 19.18; *p* = 0.0000; *F* (1, 86) = 8.94; *p* = 0.0036; and *F* (1, 85) = 7.69; *p* = 0.0068, respectively).

## 5. Discussion

The present study evaluated 88 patients with FM in detail with regard to their sociodemographic and clinical data and biographical history, focusing on the prevalence and characterization of psychological trauma across their lifespan and how it can predict the relationship with different clinical variables.

To the best of our knowledge, this is one of the few studies that has assessed the characteristics of the traumatic events suffered by a sample of women with FM in a systematic and chronological way. The results have shown that the most prevalent traumatic events categorized by age occurred during childhood and adolescence, especially in the form of physical abuse, sexual abuse, and emotional abuse, but have continued into adulthood, modifying their typology and presenting themselves mainly in the form of deaths of loved ones, harassment and physical abuse, rape, abuse, or sexual assault. These data are clinically relevant as they suggest that female FM patients are chronically and recurrently exposed to different types of stressful or vital events throughout their lives, favoring the appearance of a process of continuous retraumatization that would explain the very high percentage of PTSD diagnoses in our sample. In fact, 100% of the participants reported having suffered at least one major traumatic event at some point in their lives, and 84% had suffered one or more types of these traumatic events prior to the onset of pain. Likewise, 71.5% of the whole sample met the criteria for current PTSD, adding a further comorbidity in this complex population. These data are far higher than the prevalence data found in the general population, which range from 0.2% to 3.8% [53], and also higher than in mental disorders such as depression (48–49%) [54], bipolar disorder (4% to 40%) [55], or substance use disorder (20.67%) [56]. It is important to note that almost half of the sample (*n* = 34) was selected for the administration of the EGEP-5, an event that occurred in childhood that was the most significant traumatic event in their lives. These data, therefore, once again highlight the strong and enduring impact that childhood trauma can have on health in adulthood. Our data not only reaffirm the existence of high comorbidity between PTSD and FM but also confirm that individuals with comorbid pain and PTSD report greater pain, PTSD symptoms, depression, anxiety, disability, and opioid use than people who only have one of these conditions [57].

Regarding the results of the CTQ, our participants presented low to moderate levels of all types of childhood maltreatment, especially emotional abuse and emotional neglect, followed by sexual abuse, physical neglect, and physical abuse. These results are in line with other studies which compared FM patients to controls or patients with other pain conditions, where they found higher CTQ scores in the FM group, with emotional abuse and neglect representing the most frequent subtypes [24–27, 29, 58–60]. Other studies reported physical abuse and physical neglect as being the first [23] or the second most prevalent types of maltreatment in patients with FM [24, 25, 27, 58]. These were less frequent in our sample's CTQ scores. Interestingly, despite this, physical abuse and neglect were the most frequently reported types of maltreatment in the chronological evaluation of traumatic events in our participants. One possible explanation for this phenomenon is that, although physical maltreatment has been more present in the lives of these women, emotional abuse and neglect have had a greater impact from a psychological point of view. This hypothesis would corroborate previous research that has found not only that a lack of emotional warmth from primary caregivers is one of the greatest risk factors for the development of both physical and mental disorders in both adolescence and adulthood [61, 62], but that parental support is the most significant predictor of individual resilience [63], quality of life in adulthood [64], and mental health stability in any stage of life [65]. In fact, our results show how the presence of child abuse, in particular emotional abuse and emotional and physical neglect, predicts a poorer quality of life in the women of our sample. In addition, our results also emphasize that childhood trauma is associated with greater severity of anxious and depressive symptoms and sleep disturbances in adulthood. Therefore, regardless of the order in which the different types of maltreatment occurred, all these results underline the high prevalence of childhood maltreatment in patients affected by FM, supporting previous research that found childhood trauma is not only a risk factor for developing several mental disorders in adulthood, such as depression [66], but also for the onset and maintenance of several chronic pain conditions [61, 67]. Our data also showed that only physical neglect could explain the pain reported by patients. A possible explanation for these results could be that early exposure to traumatic events may have long-term effects on the developing nociceptive system. More specifically, physical and emotional stress could provoke alterations not only in the proper functioning of the HPA axis, favoring a hyperactivation of it, but also in the pain processing system, favoring a lack of inhibition of nociceptive stimuli by descending pain control mechanisms [68].

According to the Holmes and Rahe social readjustment scale, we found a high prevalence of major stressors that happened over the last year, compounding the prior presence of trauma-related stress and increasing its negative impact on participants' well-being. In fact, people with FM suffer from more major life stressors than patients with migraine [26], rheumatoid arthritis, and healthy controls [69], which can be explained by the increased sensitivity to stressful conditions and the high risk of retraumatization they are exposed to. A recent study has found that cumulative trauma and somatoform disorders increase the impact of FM [70], which is in line with our work. Taking into account the devastating effects of chronic stress on physical, cognitive, and mental health [71], continued exposure to trauma would increase the vulnerability of these women to the development of different pathologies, including the appearance of pain and, consequently, psychological/psychiatric comorbidities.

Although our results showed that scores on pain scales predicted the occurrence of dissociation, participants showed generally low dissociation scores on the DES scale, with the exception of the dissociation subscale, where our patients scored highly. The general low scores in dissociation are surprising, taking into account the aforementioned trauma load and high prevalence of PTSD in our sample, and also considering that dissociation is a very common phenomenon in people suffering from PTSD. A possible explanation for this could be that psychotropic medication improves dissociative symptoms by exerting an inhibitory effect on the central nervous system. However, this hypothesis needs further data because pharmacological studies available in this field are scarce and inconclusive [72]. Another explanation could be that patients with FM tend to express physically traumatic memories. The appearance of medically unexplained symptoms could occur due to the disconnection between mind and body because of the impact of traumatic experiences, followed by an amplification of subthreshold body sensations [70]. Hereby, it is interesting that our FM patients scored high on the SDQ-20, which represents somatoform dissociative disorders. Other studies has demonstrated that FM is associated with high levels of somatoform dissociation [58], in comparison with controls [70] and other functional somatic syndromes (FSSs) [73]. Furthermore, previous results suggested a direct association between childhood trauma and somatoform disorders [58, 70, 73, 74], showing childhood trauma can predict the impact and the severity of FM.

Additionally, we detected a very high prevalence of psychiatric comorbidities such as generalized anxiety disorder (87.5%), major depressive disorder (76.13%), and panic disorder (63.6%), and a lower but still important prevalence of social phobia/agoraphobia (29.5%), followed by dysthymia (11.3%), and finally obsessive-compulsive disorder (3.4%). This underlines the complexity and multidiagnostic nature of FM patients and is in line with previous literature, which reports that psychiatric comorbidity among FM, anxiety, and mood disorders is common and is a factor that can complicate medical prognosis [23,75]. Of note here, we excluded bipolar disorder, schizoaffective disorder, and schizophrenia, whereas we cannot provide prevalence data accordingly. Furthermore, we observed that FM patients received a number of psychotropic drugs, most frequently anxiolytics/hypnotics (60.9%), antidepressants (SNRI: 49.4%; SSRI: 20.6%; others: 37.9%), followed by various analgesics (paracetamol: 36.7%; anti-inflammatories: 35.6%), opioids (mainly tramadol and tapentadol, 22.7% and 9.1%, respectively) and antipsychotic medication (9.1%), indicating again the high comorbidity between FM and mental suffering. Some of the medications used, such as duloxetine, have the official indication to be prescribed for FM patients.

Our results not only confirm the high anxiety and depression scores in our sample but also that the scores obtained by the participants in the pain and fibromyalgia scales (VAS, PDI, and S-FIQ) significantly predict the presence and intensity of depressive and anxious symptomatology. This bidirectionality has been reported in previous literature showing that mental disorders, such as depression or anxiety, can be a consequence of living with chronic pain [76], as well as a history of a mental disorder being considered a risk factor for developing chronic pain [77].

Of note, 82.9% of the participants in the present study reported suicidal ideation in their history, while 32.9% reported suicide attempts. These data confirm the high prevalence of suicidal behaviors in patients suffering from chronic pain compared to the general population [9]. According to previous literature, there is a whole set of risk factors that could favor suicidal behaviors, such as psychological stress, sleep disturbances, the experience of fatigue, dizziness, and weakness, depression, female gender, and physical comorbidities like headaches and gastric diseases [9, 78–81]. Therefore, as suicidal behavior is highly prevalent due to clinical manifestations of FM and its psychiatric comorbidities, it needs to be assessed routinely in patients with FM. The same is true for sleep alteration in FM patients. Our results are in agreement with other studies, which have shown a high prevalence of sleep disturbances [6, 23, 82], which can be explained not only by pain and the direct impact of FM, but also by the presence of other axis III diagnoses, and mood and anxiety comorbidities [83, 84]. Our data revealed that 93.2% of our sample suffered from sleep alterations, which is higher than in previous studies, estimating that almost 80% of patients with FM present poor sleep [85]. Interestingly, poor sleep is strongly and dose-dependently associated with pain symptom severity in FM patients [86], at the same time as interfering with the ability to cope with pain [87], resulting in tiredness and poor quality of life [82, 88].

Finally, our sociodemographic characteristics provide insight into the high co-occurrence of various axis III diagnoses, including CFS (60.2%), confirming the overlap between both diagnoses [3,89], osteoarthritis (30.6%), spinal disc herniation (11.3%), low back pain (10.2%), and chronic migraine (10.2%). In addition, we also detected thyroid diseases (9%) and restless leg syndrome, rheumatoid arthritis, and chemical sensitivity syndrome (all of them between 5% and 6%) as somatic comorbidity. This confirms the high prevalence of physical comorbidity in FM patients and the challenges of finding adequate treatment for two or more somatic disorders [75]. Similar to previous studies, participants reported severe levels of both pain and disability [24, 27, 90, 91], which was entirely expected given that FM is characterized by severe widespread musculoskeletal pain throughout the body that ends up having repercussions in different areas of the person's life and, consequently, affects their quality of life. Moreover, the delay between the onset of pain and the clinical diagnosis of FM of the participants was, on average, almost 10 years, which may have affected their functioning and recovery processes. In fact, FM patients consider the lack of a clear explanation of the etiology and their doubts about the authenticity of the illness to be key factors in the delay in receiving a diagnosis. Both aspects are related to the lack of understanding and support from family and friends due to a lack of understanding of the nature of the illness and stigma [9], something that frequently occurs in mental disorders [92].

Several limitations have to be considered when translating our results into clinical reality. The first limitation is that we did not include a matched control group to compare our results with those of the FM group, which needs to be taken into account when interpreting and generalizing our results. Second, we did not control for possible psychiatric drug effects that could be interfering with the clinical manifestations reported by the participants. Third, the sample comprised solely female participants, so we do not know whether these results can be extrapolated to the male population. However, previous data shows that mainly female patients are affected by FM [30]. Fourth, the participants were recruited mainly from two services of our hospital, with the constraints that this entails: we might have missed FM patients who are attended by their general practitioner with lower levels of pain, clinical severity, and disability. Fifth, the method for assessing trauma varied between trauma-specific scales and an individual's chronological interview by self-report, meaning it can be affected by recall bias and an individual's subjective interpretation [93]. However, a prior study emphasizes the importance and clinical relevance of subjective memories in a recent natural human behaviour publication [94]. The authors compared in a cohort of 1196 children both objective, court-documented evidence of maltreatment and subjective reports of their childhood maltreatment histories, and found psychopathology in adulthood to be associated with subjective rather than objective measures of experience of childhood maltreatment. Furthermore, amnestic dissociation might alter the recall process; interestingly, we detected very low scores in amnestic dissociation, which further underlines the consistency of subjective recalls. Finally, we did not evaluate nonpharmacological interventions in FM, such as physical exercise or others, which can exert a positive effect on anxiety and depression.

The strengths of our work included the exhaustive evaluation of psychosocial and clinical data; a detailed evaluation of psychological trauma and its impact on participants by using validated psychometric tools; and a detailed knowledge of the chronology of traumatic events, allowing us to sequence the appearance of pain in their lives. Furthermore, the homogeneity between raters has helped to obtain data, reducing observational bias.

## 6. Conclusions

Our data highlight the complex comorbidity of FM patients with somatic disorders, psychiatric disorders, such as anxiety, depression and PTSD, highly prevalent suicidal behavior, and sleep alterations. Patients with FM, who are usually treated by rheumatologists, receive multiple psychotropic and analgesic medications, with often unsatisfactory results [95]. Our data specifically emphasize the importance of psychological trauma, especially childhood trauma, in the onset and maintenance of pain. However, close monitoring and treatment of psychological trauma are also indicated across the lifespan of FM patients. This should encourage clinicians to assess psychological trauma routinely and to include trauma-focused therapies within established multidisciplinary health care professionals, following existing FM guidelines [96]. Future research lines should test the trauma-focused intervention in FM and could also clarify the trauma-based etiology of FM in comparison to other FSSs, medically unexplained symptoms, somatic symptoms, and related disorders following the DSM-5.

## Figures and Tables

**Table 1:** Sociodemographic characteristics of the sample. Data are presented as the mean (SD) or number (%).

Variable		Obs/Freq	Mean/Percentage^*∗*^	Std. Err.	(95% Conf. Interval)	
Age		88	51.40	0.97	49.47	53.34
Education (years of studies)		88	14.05	0.41	13.23	14.87

Race	Caucasian	74	84%	0.03	0.74	0.90
	Latin	12	13.6%	0.03	0.07	0.22
	Other	2	2.2%	0.01	0.005	0.08

Relationship status	Single	16	18.1%	0.04	0.11	0.27
	Married/in a couple	69	78.4%	0.04	0.68	0.85
	Widowed	3	3.4%	0.01	0.01	0.10

Employment status	Full-time employment	12	13.6%	0.03	0.07	0.22
	Part-time employment	5	5.6%	0.02	0.02	0.13
	Sick leave	31	35.2%	0.05	0.25	0.45
	Unable to work due to mental health problems	6	6.8%	0.02	0.03	0.14
	Unable to work due to other reasons	17	19.3%	0.04	0.12	0.29
	Unemployed	31	14.7%	0.03	0.08	0.23
	Others	3	3.4%	0.01	0.01	0.10
	Student	1	1.1%	0.01	0.001	0.07

Obs/Freq, Number of cases observed/Frequency; Std. Error, Standard Error; Conf, Confidence ^*∗*^Age and education data are presented as means. The rest of the variables are presented as percentages.

**Table 2:** Clinical characteristics of the sample. Data are presented as the mean (SD) and/or number (%).

Variable		Obs/Freq	Mean/Percentage^*∗*^	Std. Err.	[95% Conf. Interval]	
MINI	GAD	77	87.5%	0.03	0.78	0.92
	MDD	67	76.13%	0.04	0.65	0.83
	Panic disorder	56	63.6%	0.05	0.52	0.73
	Social phobia/Agoraphobia	26	29.5%	0.04	0.20	0.40
	Dysthymia	10	11.3%	0.03	0.06	0.19
	OCD	3	3.4%	0.01	0.01	0.10

HADS	HADS-D	88	12.04	0.46	11.12	12.96
	HADS-A	88	14.34	0.39	13.55	15.12
AIS		88	14.13	0.52	13.10	15.17
SWLS		87	11.78	0.49	10.79	12.76
VAS pain		88	6.57	0.23	6.11	7.03
PDI		88	46.12	1.21	43.71	48.53
Onset of pain		86	35.44	1.29	32.87	38.02
Diagnosis of fibromyalgia		87	44.55	0.92	42.73	46.37
S-FIQ		87	55.58	1.06	53.47	57.69
Suicidal thinking		73	82.9%	0.04	0.73	0.89

Suicide attempt	28	32.9%	0.05	0.23	0.43	
	Violent	4	4.5%	0.02	0.01	0.11
	No violent	24	27.2%	0.04	0.18	0.37
	Severe	12	13.6%	0.03	0.078	0.22
	Not severe	16	18.1%	0.04	0.11	0.27

Diagnostics axis III	CFS	53	60.2%	0.05	0.49	0.70
	Osteoarthritis	27	30.6%	0.04	0.21	0.41
	Spinal disc herniation	10	11.3%	0.03	0.06	0.19
	Chronic migraine	9	10.2%	0.03	0.05	0.18
	Low back pain	9	10.2%	0.03	0.05	0.18
	Thyroid diseases	8	9%	0.03	0.04	0.17
	Rheumatoid arthritis	6	6.8%	0.02	0.03	0.14
	Restless legs syndrome	5	5.6%	0.02	0.02	0.13
	Chemical sensitivity syndrome	4	4.5%	0.02	0.01	0.11

Pharmacological treatment	Anxiolytics/Hypnotics	53	60.9%	0.05	0.50	0.70
	SNRI	43	49.4%	0.05	0.38	0.59
	SSRI	18	20.6%	0.04	0.13	0.30
	Other antidepressants	33	37.9%	0.05	0.28	0.48
	Opioids+ Tramadol Codeine Morphine Fentanyl Oxycodone Buprenorphine Tapentadol	32 20 1 1 1 2 1 8	36.7% 22.7% 1.1% 1.1% 1.1% 2.3% 1.1% 9.1%	0.05 0.04 0.01 0.01 0.01 0.01 0.01 0.03	0.27 0.14 −0.01 −0.01 −0.01 −0.01 −0.01 0.03	0.47 0.32 0.03 0.03 0.03 0.06 0.03 0.16
	Paracetamol	32	36.7%	0.05	0.27	0.47
	Anti-inflammatory drugs	31	35.6%	0.05	0.26	0.46
	Anticonvulsants	18	20.6%	0.04	0.13	0.30
	Antipsychotics	8	9.1%	0.03	0.04	0.17

Obs/Freq : Number of cases observed/Frequency; Std. Error, Standard Error; Conf., Confidence; MINI : MINI Neuropsychiatric International Interview; MDD : Major Depressive Disorder; GAD : Generalized Anxiety Disorder; OCD : Obsessive-Compulsive Disorder; HADS : Hospital Anxiety and Depression Scale; HADS-D : Hospital Anxiety and Depression Scale-Depression; HADS-A : Hospital Anxiety and Depression Scale-Anxiety; AIS : Athens Insomnia Scale; SWLQ : Satisfaction With Life Questionnaire; VAS pain, Visual Analogue Scale for pain; PDI : Pain Disability Index; S-FIQ : Fibromyalgia Impact Questionnaire-Spanish validation; CFS : Chronic Fatigue Syndrome; SSRI : Selective Serotonin Reuptake Inhibitor; SNRI : Serotonin-Norepinephrine Reuptake Inhibitor. ^*∗*^Scales data are presented as means. The rest of the variables are presented as percentages. + also in combination.

**Table 3:** Clinical variables of psychological trauma. Data are presented as the mean (SD).

Variable		Obs/Freq	Mean/Proportion	Std. Err.	(95% Conf. Interval)	
CTQ	Total	88	49.97	2.11	45.78	54.17
	Emotional A	88	12.10	0.64	10.81	13.39
	Physical A	88	8.19	0.54	7.11	9.27
	Sexual A	88	8.76	0.60	7.55	9.96
	Emotional N	88	12.72	0.63	11.47	13.97
	Physical N	88	7.51	0.30	6.89	8.12

EGEP-5	PTSD	63	0.71	0.04	0.61	0.80
	No PTSD	24	0.27	0.04	0.18	0.37
	NA	1	0.01	0.01	0.001	0.07
SUD		87	8.23	0.26	7.71	8.75

DES	Total	86	24.72	1.34	22.05	27.39
	Amnesia	86	11	1.20	8.61	13.38
	Depersonalization Derealization	86	19.47	1.63	16.22	22.72
	Dissociation	86	36.26	1.86	32.55	39.97

SDQ-20	Number of symptoms	86	4.76	0.30	4.16	5.37
	Total score	86	39.45	1.11	37.22	41.67

Holmes and Rahe	Number of events	88	7.64	0.42	6.80	8.49
	Total scores	88	224.52	13.16	198.34	250.69

Obs/Freq : Number of cases observed/Frequency; Std. Error, Standard Error; Conf., Confidence; CTQ : Childhood Trauma Questionnaire; Emotional A : Emotional Abuse; Physical A : Physical Abuse; Sexual A : Sexual Abuse; Emotional N : Emotional Neglect; Physical N : Physical Neglect; EGEP-5 : Global Assessment of Post-traumatic Stress Questionnaire-5; PTSD : Post-traumatic Stress Disorder; NA : Not Applicable; SUD : Subjective Unit of Disturbance; DES : Dissociative Experiences Scale; SDQ-20 : Somatoform Dissociation Questinnaire-20; Number of symptoms: Number of symptoms with unknown cause; Holmes and Rahe : Holmes and Rahe Social Readjustment Scale.

**Table 4:** Linear regression results.

Variables^*∗*^	Obs	F and prob > F	R-squared and Adj R-squared	Linear regression equation	t and P > (t)	(95% Conf. Interval)	
SWLS (Y) CTQ-T (X)	87	*F* (1, 85) = 8.27; *p*=0.0051	*R * ^2^ = 0.0887 Adj *R*^2^ = 0.0780	^*Y* = −0.069 × 15.25	*t* = −2.88; *p*=0.005	−0.117	−0.021
SWLS (Y) CTQ-EA (X)	87	*F* (1, 85) = 6.30; *p*=0.0140	*R * ^2^ = 0.0690 Adj *R*^2^ = 0.0580	^*Y* = −0.198 × 14.18	*t* = −2.51; *p*=0.014	−0.355	−0.411
SWLS (Y) CTQ-EN (X)	87	*F* (1, 86) = 12.94; *p*=0.0005	*R * ^2^ = 0.1321 Adj *R*^2^ = 0.1219	^*Y* = −0.283 × 15.40	*t* = −3.60; *p*=0.014	−0.440	−0.126
SWLS (Y) CTQ-PN (X)	87	*F* (1, 85) = 6.10; *p*=0.0155	*R * ^2^ = 0.0669 Adj *R*^2^ = 0.0560	^*Y* = −0.410 × 14.87	*t* = −2.47; *p*=0.016	−0.741	−0.080
HAD-D (Y) VAS (X)	88	*F* (1, 86) = 3.99; *p*=0.0489	*R * ^2^ = 0.0443 Adj *R*^2^ = 0.0332	^*Y* = 0.419 × 9.28	*t* = 2.00; *p*=0.049	0.001	0.837
HAD-A (Y) AS (X)	88	*F* (1, 86) = 10.08; *p*=0.0021	*R * ^2^ = 0.1049 Adj *R*^2^ = 0.0945	^*Y* = 0.547 × 10.74	*t* = 3.18; *p*=0.002	0.204	0.889
AIS (Y) VAS (X)	88	*F* (1, 86) = 19.18; *p*=0.000	*R * ^2^ = 0.1823 Adj *R*^2^ = 0.1728	^*Y* = 0.952 × 7.87	*t* = 4.38; *p*=0.000	0.520	1.385
DES-T (Y) VAS (X)	86	*F* (1, 86) = 4.31; *p* = 0.0410	*R * ^2^ = 0.0488 Adj *R*^2^ = 0.0375	^*Y* = 1.262 × 16.46	*t* = 2.08; *p*=0.041	0.053	2.47
DES-D (Y) VAS (X)	86	*F* (1, 84) = 4.30; *p*=0.0412	*R * ^2^ = 0.0487 Adj *R*^2^ = 0.0374	^*Y* = 1.750 × 24.81	*t* = 2.07; *p*=0.041	0.071	3.43
HAD-D (Y) PDI (X)	88	*F* (1, 86) = 31.14; *p*=0.0000	*R * ^2^ = 0.2658 Adj *R*^2^ = 0.2573	^*Y* = 0.197 × 2.94	*t* = 5.58; *p*=0.000	0.127	0.267
HAD-A (Y) PDI (X)	88	*F* (1, 86) = 22.58; *p*=0.0000	*R * ^2^ = 0.2079 Adj *R*^2^ = 0.1987	^*Y* = 0.147 × 7.52	*t* = 4.75; *p*=0.000	0.086	0.209
AIS (Y) PDI (X)	88	*F* (1, 86) = 8.94; *p*=0.0036	*R * ^2^ = 0.0941 Adj *R*^2^ = 0.0836	^*Y* = 0,131 × 8,07	*t* = 2.99; *p*=0.004	0.044	0.218
SWLS (Y) PDI (X)	87	*F* (1, 85) = 8.69; *p*=0.0041	*R * ^2^ = 0.0927 Adj *R*^2^ = 0.0821	^*Y* = -0.122 × 17.44	*t* = −2.95; *p*=0.004	−0.205	−0.039
DES-T (Y) PDI (X)	86	*F* (1, 84) = 10.50; *p*=0.0017	*R * ^2^ = 0.1111 Adj *R*^2^ = 0.1005	^*Y* = 0.361 × 8.10	*t* = 3.24; *p*=0.002	0.139	0.582
DES-A (Y) PDI (X)	86	*F* (1, 85) = 6.27; *p*=0.0142	*R * ^2^ = 0.0695 Adj *R*^2^ = 0.0584	^*Y* = 0.2551 × -0.74	*t* = 2.50; *p*=0.014	0.052	0.457
DES-DP (Y) PDI (X)	86	*F* (1, 85) = 6.29; *p*=0.0140	*R * ^2^ = 0.0697 Adj *R*^2^ = 0.0586	^*Y* = 0.3481 × 3.45	*t* = 2.501 *p*=0.014	0.072	0.624
DES-D (Y) PDI (X)	86	*F* (1, 85) = 7.56; *p*=0.0073	*R * ^2^ = 0.0826 Adj *R*^2^ = 0.0716	^*Y* = 0.4331 × 16.37	*t* = 2.75 *p*=0.007	0.119	0.744
HAD-D (Y) S-FIQ (X)	87	*F* (1, 85) = 12.39; *p*=0.0007	*R * ^2^ = 0.1272 Adj *R*^2^ = 0.1170	^*Y* = 0.1531 × 3.60	*t* = 3.52 *p*=0.001	0.066	0.240
HAD-A (Y) S-FIQ (X)	87	*F* (1, 85) = 27.16; *p*=0.0000	*R * ^2^ = 0.2422 Adj *R*^2^ = 0.2333	^*Y* = 0.1741 × 4.79	*t* = 5.21 *p*=0.000	0.107	0.240
AIS (Y) S-FIQ (X)	87	*F* (1, 85) = 7.69; *p*=0.0068	*R * ^2^ = 0.0830 Adj *R*^2^ = 0.722	^*Y* = 0.137 × 6.64	*t* = 2.77 *p*=0.007	0.038	0.235
SWLS (Y) S-FIQ (X)	86	*F* (1, 85) = 8.08; *p*=0.0056	*R * ^2^ = 0.0877 Adj *R*^2^ = 0.769	^*Y* = −0.136 × 19.24	*t* = −2.84 *p*=0.006	−0.231	−0.040
DES-T (Y) S-FIQ (X)	85	*F* (1, 83) = 11.83; *p*=0.0000	*R * ^2^ = 0.1247 Adj *R*^2^ = 0.114	^*Y* = 0.440 × 0.488	*t* = −3.44 *p*=0.001	0.185	0.695
DES-A (Y) S-FIQ (X)	85	*F* (1, 83) = 8.61; *p*=0.0043	*R * ^2^ = 0.0940 Adj *R*^2^ = 0.831	^*Y* = 0.342 × −7.860	*t* = 2.93 *p*=0.004	0.110	0.574
DES-D (Y) S-FIQ (X)	85	*F* (1, 83) = 5.08; *p*=0.0268	*R * ^2^ = 0.0577 Adj *R*^2^ = 0.046	^*Y* = 0.367 × −0.792	*t* = 2.25 *p*=0.027	0.043	0.691
DES-D (Y) S-FIQ (X)	85	*F* (1, 83) = 10.25; *p*=0.0019	*R * ^2^ = 0.1099 Adj *R*^2^ = 0.099	^*Y* = 0.575 × 4.56	*t* = 3.20 *p*=0.002	0.217	0.933
VAS (Y) CTQ-PN (X)	88	*F* (1, 86) = 4.12; *p*=0.0455	*R * ^2^ = 0.0457 Adj *R*^2^ = 0.0346	^*Y* = −0.161 × 7.78	*t* = -2.03 *p*=0.046	−0.003	9.058

^*∗*^Linear regression equation for predicting Y from *X* in two ways. CTQ-EA : CTQ Emotional Abuse; CTQ-EN : CTQ Emotional Neglect; CTQ-Physical Neglect; DES-T : DES Total; DES-D : DES Dissociation; DES-A : DES Amnesia; DES-D : DES Depersonalization; HADS-DP : Hospital Anxiety and Depression Scale-Depression; HADS-A : Hospital Anxiety and Depression Scale-Anxiety; VAS : Visual Analogue Scale for pain; AIS : Athens Insomnia Scale; PDI : Pain Disability Index; S-FIQ : Fibromyalgia Impact Questionnaire-Spanish validation; SWLS : Satisfaction With Life Scale.

## Data Availability

Data are available on request due to privacy/ethical restrictions.
